# Identification of heavy metal-mobilizing bacteria and revealing of their mechanisms for bioremediation of Pb–Cd co-contaminated soils with *Brassica juncea*

**DOI:** 10.1128/spectrum.01964-25

**Published:** 2026-03-18

**Authors:** Qiyu Zhang, Zhenyu Zhang, Tianci Wu, Shiying Li, Xueqiang Zhu, Haitao Liu, Xia Xue, Ying Jiang

**Affiliations:** 1 College of Resources and Environment, Henan Agricultural University 70573, Zhengzhou, People's Republic of China; 2 State Key Laboratory of High-Efficiency Production of Wheat-Maize Double Cropping, Henan Agricultural University 70573, Zhengzhou, People's Republic of China; 3 Henan Key Laboratory for Helicobacter pylori and Digestive Tract Microecology, The Fifth Affiliated Hospital of Zhengzhou University 12636, Zhengzhou, People's Republic of China; Università degli Studi di Napoli Federico II, Naples, Italy

**Keywords:** *Brassica juncea*, heavy metal-mobilizing bacteria, heavy metal transporter genes, Pb–Cd co-contamination, soil bioremediation

## Abstract

The effective strategies for soil remediation in lead (Pb) and cadmium (Cd) co-contaminated areas are critical for environmental restoration. This study aimed to develop a microbial-plant remediation approach by combining a Pb–Cd mobilizing bacterium with the heavy metal-accumulating plant *Brassica juncea*, elucidating the underlying mechanisms of microbial-assisted phytoremediation. A Pb–Cd mobilizing bacterial strain, HM-18, was isolated from mine tailings and had been identified as *Microbacterium foliorum*. Soil microcosm experiments with four treatments were conducted to evaluate its remediation efficiency, including control (CK), only HM-18 inoculation (B), Pb/Cd contamination (HM), and combined HM-18 inoculation with Pb/Cd contamination (HM+B). We found that the HM+B treatment significantly reduced soil pH by 2.81%, while increasing soil enzyme activities, including catalase (12.16%), alkaline phosphatase (3.20%), and sucrase (21.46%). Also, inoculation with HM-18 alleviated heavy metal stress in *B. juncea* significantly by enhancing biomass, photosynthetic efficiency, and antioxidant enzyme activities, thereby mitigating oxidative damage. Furthermore, the expression of key metal transporter genes was increased with HM-18 inoculation, while *HMA3* was decreased, which promotes the translocation of Pb and Cd from roots to shoots. The accumulation of Pb and Cd in shoots increased by 72.38% and 27.61%, respectively, with root accumulation rising by 76.46% (Pb) and 56.14% (Cd). This work confirmed that this synergistic system enhanced heavy metal removal through multiple pathways, including increased metal bioavailability, improved root morphology, enhanced photosynthetic capacity, strengthened stress resistance, and regulated transporter activity. These findings offer valuable microbial resources and a theoretical basis for the development of sustainable soil bioremediation strategies.

**IMPORTANCE:**

This work advances the development of sustainable solutions for remediating soils contaminated with harmful heavy metals like lead (Pb) and cadmium (Cd), which pose significant risks to environmental and human health. By developing a microbial-plant remediation strategy that leverages the synergistic effects of a mobilizing bacterium and a metal-accumulating plant, this research improves our ability to effectively detoxify contaminated soils. The insights gained into the underlying mechanisms provide a valuable foundation for designing efficient, environmentally friendly bioremediation strategies. Generally, this investigation contributes to safeguarding ecosystems, promoting soil restoration, and supporting sustainable land use in areas affected by heavy metal pollution.

## INTRODUCTION

Heavy metals, characterized by their toxicity and non-biodegradability, pose significant threats to soil ecosystems. They can enter and accumulate in the human digestive system through the food chain, resulting in serious risks to global health (1). Notably, the heavy metal contamination in soils often involves the co-existence of multiple metals, making it more complex and widespread, which defines the complexity of soil heavy metal compound pollution (2). Cadmium (Cd) and lead (Pb) are common soil contaminants (3), and their excessive accumulation can induce physiological, ultrastructural, and biochemical alterations in plants, leading to growth inhibition, metabolic disorders, and consequently crop yield and quality reductions (4, 5). Therefore, it is valuable to develop a reliable, environmentally friendly, and economically viable strategy to control and mitigate heavy metal pollution across broad soil ecosystems (6).

Compared to conventional physical and chemical remediation techniques, phytoremediation is regarded as an environmentally friendly, non-invasive, energy-efficient, and cost-effective approach (7, 8). Indian mustard [*Brassica juncea* (L.) Czern., Brassicaceae, the crucifer family] shows a high phytoremediation potential for heavy metal-contaminated soils (9). However, the low biomass and limited heavy metal accumulation capacity mainly limit the efficacy of phytoextraction (10). In contrast, few soil microbes gain genetic resistance to heavy metals and modulate their bioavailability through interactions (11–13). To date, integrated microbial approaches and phytoremediation have gained increasing attention due to their high efficiency and safety in soil bioremediation. Moreover, the plant-microbial combination enhances reductive activity and helps overcome challenges related to low efficiency and lengthy bioremediation cycles (14). Soil microorganisms alter the chemical forms of heavy metals, while plant roots absorb heavy metals in ionic form and transfer them from soil to plants through processes such as absorption, extraction, volatilization, and stabilization, thereby achieving removal (14).

Rhizospheric and endophytic bacteria affect the availability of heavy metals in soil, thereby affecting their uptake and accumulation by plants (15). Sheng et al. reported that specific endophytic bacteria can activate heavy metals and increase their bioavailability by producing organic acids, iron chelators, and other bioactive compounds (16). *Endophyte Bacillus* SLS18 enhanced the heavy metal carrying capacity of sorghum (17). A previous study found that the Cd content of *B. juncea* roots greatly increased following inoculation with rhizosphere bacteria (18). These bacteria can thereby reduce the heavy metal stress effects on plants and promote plant growth by producing various phytohormones and biomolecules (19). Kamran et al. identified plant growth-promoting bacteria (PGPB) (*Pseudomonas putida*) from heavy metal-contaminated soil and found that this bacterium promoted the growth of *Eruca sativa* and increased Cd uptake upon inoculation (20). Furthermore, bacterial species resistant to Pb and Cd, isolated from heavy metal-contaminated soils, increased both the biomass and the Pb and Cd uptake of ornamental cabbage (21).

As the part directly interacting with the soil, plant roots bear the brunt of toxic ionic stress (22). Heavy metal contamination often induces changes in root morphological characteristics, including parameters such as root length, surface area, and microstructure, which in turn affect nutrient absorption and aboveground growth. Lu et al. found that excessive Cd significantly reduced the root growth of peanuts, suggesting that root morphological parameters can serve as reliable indicators of Cd toxicity (23). Furthermore, previous studies have found that both Cd and Pb severely disturb cell division, mineral nutrient uptake and transport, biosynthesis of photosynthetic pigments, photosynthetic and respiratory processes, protein synthesis, enzyme activities, and reactive oxygen species (ROS) metabolism. These disruptions lead to the inhibition of growth, development, and yield of various crops worldwide (24–26).

Soil enzyme activities are important biological indicators of soil health and quality because enzymes are responsive to metal stress and play a direct role in soil processes related to the cycling of carbon (C), nitrogen (N), and phosphorus (P) (27–30). Common soil enzymes, such as β-glucosidase, urease, protease, and phosphatase, are essential for nutrient utilization and cycling within the soil ecosystem (31). To counteract oxidative damage, plants have developed natural antioxidant defense systems that include the production of antioxidants and scavenging mechanisms to regulate intracellular ROS levels (32). This defense system comprises both enzymatic and non-enzymatic components (33, 34). Among these, the antioxidant enzymes play a critical role in responding to metal-induced toxicity. For instance, enzymes such as guaiacol peroxidase (POD), ascorbate peroxidase (APX), superoxide dismutase (SOD), and catalase (CAT) are instrumental in maintaining cellular redox homeostasis at safe levels (35–37). It has also been suggested that the uptake of heavy metals by plant roots is closely linked to the transcription of heavy metal-transporting genes. Accordingly, this study screened bacterial strains with Pb- and Cd-mobilizing capabilities. By conducting a soil microcosm experiment, we investigated the synergistic effects of Pb–Cd mobilizing bacteria and *B. juncea* in remediating co-contaminated soils, while elucidating the underlying physiological and biochemical mechanisms. Our work aims to address the gap in understanding the mechanisms of combined microbial-plant remediation in Pb- and Cd-contaminated soils.

## MATERIALS AND METHODS

### Strain screening and identification

#### Isolation of Pb- and Cd-tolerant bacteria

A vigorously growing plant was selected from a mining tailing in Xinxiang, Henan Province. The heavy metal concentrations in the tailing were as follows: 38.9 mg·kg^−1^ Pb, 0.23 mg·kg^−1^ Cd, 70.8 mg·kg^−1^ Hg, 8.53 mg·kg^−1^ As, 129 mg·kg^−1^ Cu, 82 mg·kg^−1^ Ni, and 16 mg·kg^−1^ Zn. Rhizosphere soil was collected using sterile brushes (38). A 10 g soil sample was weighed and transferred into a conical flask containing 90 mL of sterile water and an appropriate number of glass beads. The flask was then placed in a shaker and agitated at 180 × *g* for 30 min at room temperature and then allowed to settle for 20 min. The supernatant was subjected to serial dilution (10^−2^ to 10^−5^), and 50 μL aliquots from each dilution were spread onto Luria-Bertani (LB) agar plates. Three replicate plates were prepared per dilution. All plates were incubated at 28°C for 48 h. Following colony formation, distinct, well-isolated, and vigorously growing single colonies were selected and streaked onto fresh LB agar plates for purification. The purified strains were stored at 4°C for subsequent use.

The tolerance of each strain to Cd and Pb was evaluated by the minimum inhibitory concentration (MIC) method, as described in Abou-Shanab et al. (39). CdCl₂ and PbCl₂ were dissolved in deionized water, then filtered through a sterile 0.45 μm membrane. The solutions were adjusted to a final volume of 1 L in a volumetric flask, yielding concentrations of 1.5 g·L^−^¹ for Cd and 2.0 g·L^−^¹ for Pb. These Cd and Pb solutions were added to De Man, Rogosa, and Sharpe (MRS) solid medium to prepare media containing final metal ion concentrations (mg·L^−^¹) of 10, 30, 50, 100, 150, and 200 for Cd, and 500, 1,000, 1,500, 2,000, 2,500, and 3,000 for Pb. A 20 μL aliquot of bacterial suspension was inoculated into each MRS medium containing the metal ions (three biological replicates per group) using a micropipette, and the plates were incubated at 37°C for 24 h. In this study, an MRS medium without metal ions served as the control, and bacterial growth was observed and recorded.

#### Validation of Pb and Cd mobilization capacity by Pb- and Cd-tolerant bacteria

The bacterial inoculum was prepared using 20-h log-phase cells. The medium composition was identical to the selection medium described above and supplemented with 200 mg·L^−1^ PbCO₃ and 100 mg·L^−1^ CdCO₃. Triplicate 250-mL Erlenmeyer flasks containing 100 mL of media (autoclaved at 121°C for 30 min) were inoculated with 1 mL of the bacterial culture (~10⁸ CFU·mL^−^¹). Control setups included uninoculated media amended with Pb/Cd and inoculated media without Pb/Cd to assess abiotic effects on Pb/Cd solubility and the influence of Pb/Cd on pH. Both control and experimental flasks were autoclaved at 121°C for 30 min and incubated at 28°C on a rotary shaker at 150 × *g*. After 48 h, the cultures were filtered through a 0.22-μm Millipore filter and analyzed for pH and optical density at 600 nm (OD₆₀₀). The remaining culture was used to determine water-soluble Pb and Cd. This depleted medium was centrifuged at 925 × *g* for 20 min at 6°C, then filtered through a 0.22-μm membrane. The concentrations of Pb and Cd in the supernatant were measured using an atomic absorption spectrophotometer (Model TAS-986, Beijing, China).

#### Identification of Pb–Cd mobilizing bacteria

Purified bacterial isolates from the culture plate using an inoculation loop. They were then characterized through various methods, including morphological analysis (assessing size, shape, opacity, margin, consistency, and pigmentation), as well as physiological and biochemical tests, including the Methyl Red test, citrate utilization, nitrate reduction, gelatin hydrolysis, sugar fermentation, and starch hydrolysis. The 16S rRNA gene was amplified with the universal primers 27 F (5′-GAGTTTGATCACTGGCTCAG-3′) and 1492 R (5′-TACGGCTACCTTGTTACGACTT-3′) (40). The PCR products were purified and sequenced by Sangon Biotech (Shanghai) Co., Ltd. The gene sequence was analyzed using the BLAST and EzTaxon services via the NCBI database. Finally, the phylogenetic tree of the 16S rRNA gene was constructed by MEGA X using the neighbor-joining algorithm (41).

#### Identification of plant growth-promoting traits of Pb–Cd mobilizing bacteria

The Pb–Cd mobilizing bacteria were incubated in 50 mL of potassium solubilizing medium at 28°C for 72 h with shaking at 180 × *g*. To remove any insoluble material, the fermentation broth was first centrifuged at 500 × *g* for 10 min. The supernatant was then collected and further centrifuged at 10,000 × *g* for 5 min. The experiment was conducted in triplicate (biological replicates), with uninoculated medium serving as the control. Flame spectrophotometry was used to measure the potassium content in the supernatant (42).

The production of siderophores by the bacteria was assessed using the chrome azurol-S (CAS) assay method (43, 44). Cells and supernatants were separated by centrifugation at 9,000 × *g* for 10 min. Then, 1.0 mL of the supernatant was mixed with 1.0 mL of CAS assay solution (43). A blank control was prepared by combining 1.0 mL of the CAS assay solution with 1.0 mL of uninoculated medium used for bacterial culture. The absorbance was measured at 630 nm one hour after mixing, and the results were compared to the optical density (O.D.) of the reference.

### Soil microcosm experiment on combined remediation technology using Pb–Cd mobilizing bacteria and *B. juncea*

#### Experimental design

Soil samples were collected from the experimental field at Xuchang Campus of Henan Agricultural University (0–20 cm depth), with the following physicochemical properties: total nitrogen 1.02 g·kg^−1^, organic matter 13.67 g·kg^−1^, available nitrogen 66.21 mg·kg^−1^, available potassium 159.75 mg·kg^−1^, available phosphorus 8.51 mg·kg^−1^, and pH 8.51. The soil samples were air-dried to remove moisture, and all stones, plant debris, and other impurities were carefully removed. The soil was then passed through a 2 mm mesh to ensure uniform particle size (45). After thorough mixing, the soil was artificially contaminated with Pb at 800 mg·kg^−1^ and Cd at 80 mg·kg^−1^, using PbCO_3_ and CdCO_3_, respectively. The contaminated soil was mixed again to ensure even distribution of the metals and then left for 60 days to allow for aging and stabilization. For preparation of the bacterial inoculum, pure cultures of the HM-18 strain were inoculated into sterile liquid LB medium and incubated with shaking at 30°C at a rotation speed of 180 × *g*. Before inoculation, the optical density of the bacterial suspension was measured to ensure an OD₆₀₀ of approximately 0.8 (46).

A pot experiment with 10 biological replicates was arranged in a randomized design at the Henan Agricultural University. The four treatments were CK (control, no heavy metals or bacteria), B (inoculated with *Microbacterium foliorum* only, no heavy metals), HM (heavy metals only, no bacteria), and HM+B (heavy metals with *M. foliorum* inoculation). The soils for the HM and HM+B treatments were spiked with 800 mg·kg^−1^ Pb and 80 mg·kg^−1^ Cd. Each treatment was represented by 10 pots, with each pot measuring 16.5 cm in diameter and 10.2 cm in height, and filled with 650 g of soil. Uniformly healthy *B. juncea* seedlings were selected and transplanted into individual pots, one seedling per pot. Specifically, for treatments requiring inoculation (B and HM+B), seedlings were immersed in an *M. foliorum* suspension for 2 h before transplanting into the prepared pots. Seedlings soaked in sterile water served as the non-inoculated control. On the seventh day after sowing, 40 mL of *M. foliorum* suspension with an optical density at 600 nm (OD₆₀₀) of approximately 0.8 was slowly irrigated into the soil around the mustard roots (46). During irrigation, the pots were gently shaken to ensure the bacterial suspension fully contacted and mixed with the soil. Reinoculation was conducted once a week thereafter. Bacterial cells used for preparing the suspension were all cultured in LB medium. For the non-inoculated group (CK and HM), an equal volume (40 mL) of sterilized LB medium was irrigated synchronously during each inoculation event to serve as the control for the irrigation step. The control soil was left untreated without adding metals or inoculating bacteria. The soil microcosm experiment was conducted in a greenhouse under controlled conditions (16/8 h light/dark cycle, 60% ± 5% relative humidity, 30°C). Plants were watered daily to maintain soil moisture at 60%–70% of field capacity. Sampling was performed after 60 days of *B. juncea* cultivation. A total of 10 biological replicates were maintained per treatment.

#### Plant biomass measurement

At the conclusion of the 60-day soil microcosm experiment, plant samples were collected and thoroughly washed with distilled water. The seedlings were separated into roots and shoots using clean scissors. Roots were immersed in a 1 mM EDTA-Na_2_ solution for 15 min, then rinsed three times with deionized water to remove surface-bound heavy metals. Fresh samples were immediately frozen in liquid nitrogen and stored at −80°C for further analysis. The lengths of both shoots and roots were measured with a ruler (minimum scale of 0.1 cm), and their fresh weights were recorded using a balance with 0.0001 g precision. Dry weights were determined after drying the samples in an oven at 105°C for 30 min, followed by drying at 70°C until they reached a constant weight. Additionally, a 50 g soil sample was collected from each pot after thoroughly mixing the soil, and the soil properties were analyzed following air drying.

#### Pb and Cd concentrations and physicochemical properties of the soil

After a 60-day incubation period, soil samples were collected from all treatment pots. Bioavailable Pb and Cd in the soil were determined according to the procedure of Goncalves da Silva et al. (47). Briefly, 5 g of soil sample was shaken in 25 mL DTPA-extraction solution for 2 h at 180 × *g* in a reciprocal shaker. The resulting suspension was filtered through filter papers (Whatman 202), and the concentrations of Pb and Cd in the filtrate were determined by an atomic absorption spectrophotometer (ZEEnit700, Jena, Germany). Additionally, the following soil properties were measured: pH, soil organic matter (SOM), total nitrogen (TN), available nitrogen (AN), available phosphorus (AP), and available potassium (48–51).

#### Soil enzyme activity assay

The activities of soil urease (S-URE), catalase (S-CAT), sucrase (S-INV), and alkaline phosphatase (S-ALP) were quantified using 96-well enzyme-linked immunosorbent assay kits following the Double-Antibody Sandwich method, with detection ranges of 30–1,200 IU/L, 0.3–9 U/mL, 22–800 U/L, and 1.5–60 IU/L, respectively. Briefly, microplates were pre-coated with purified antibodies specific to each enzyme to generate solid-phase antibodies. Corresponding enzymes extracted from soil samples and horseradish peroxidase-conjugated antibodies against the target enzymes were sequentially added to the microplate wells, facilitating the formation of antibody-antigen-enzyme-labeled antibody immunocomplexes. After thorough washing to remove unbound components, 3,3′,5,5′-tetramethylbenzidine substrate was added to initiate color development. The absorbance (optical density, OD) was measured at 450 nm using a microplate reader, and the activity concentration of each enzyme was calculated by referencing a standard calibration curve constructed from serial dilutions of respective enzyme standards (52–55).

#### Root morphology measurement

Three root samples of *B. juncea* were randomly collected from each treatment. The fresh roots were scanned using an automated root scanner (Epson V700 PHOTO, Japan), and the root images were analyzed with the root image analysis software WinRHIZO 2003b to obtain the total root length (RL), root surface area (SA), number of root tips (RT), and average root diameter (RD). Based on the measured average diameter, the total root length and root surface area were classified into four diameter classes at 0.5 mm intervals: class I (RD 0–0.5 mm); class II (RD 0.5–1.0 mm); class III (RD 1.0–1.5 mm); and class IV (RD > 1.5 mm) (56).

#### Measurement of plant photosynthetic parameters and chlorophyll

The net photosynthetic rate (Pn), stomatal conductance (Gs), intercellular CO₂ concentration (Ci), and transpiration rate (Tr) of the fully expanded fresh leaves were measured 1 day prior to harvest using a Li-6400 portable photosynthetic system (LI-COR, Lincoln, USA). A portable SPAD-502 chlorophyll meter (Model SPAD- 502, Konica Minolta, Osaka, Japan) was used to assess the SPAD chlorophyll index of the leaves. The relative chlorophyll content was determined by measuring the absorption rates of the leaves in the near-infrared region (940 nm) and the red region (650 nm). Each measurement was repeated five times at each site, and the average value was recorded.

During sample collection, 0.3 g of fresh leaf tissue was immediately immersed in 25 mL of 95% ethanol and kept in darkness until the leaf color had completely faded. The absorbance of the resulting extracts was measured at 470, 649, and 665 nm using a UV/VIS spectrophotometer (Model L5, Shanghai Yidian Analysis Instrument Co., Ltd., China), with 95% ethanol serving as the blank. Subsequently, the contents of chlorophyll a (Chl a), chlorophyll b (Chl b), and carotenoids in *B. juncea* leaves were calculated (57).

#### Measurement of antioxidant compounds and antioxidant enzyme activities

Root samples (0.1 g) were mixed with 1 mL of extraction buffer, ground in an ice bath, and then centrifuged. The supernatant was transferred to a microcentrifuge (Eppendorf) tube for subsequent biochemical assays. The activities of CAT, SOD, POD, and APX were determined spectrophotometrically using commercial assay kits (Suzhou Grace Biotechnology Co., Ltd., Suzhou, China).

Root samples (0.5 g) were ground with 5 mL of 5% trichloroacetic acid solution in a pre-cooled mortar until thoroughly homogenized. The homogenate was then centrifuged at 10,000 × *g* for 10 min at 4°C (H2050R, XIANGYI, China). An aliquot of the supernatant was used to determine the contents of malondialdehyde (MDA) and various antioxidant substances. MDA content was determined following the method of Heath and Packer (58). Ascorbate (AsA) levels were determined using the protocol described by Zhang and Kirkham (59). Glutathione (GSH) content was determined using the procedure of Guri (60). Non-protein thiols (NPT) were determined according to Zhang et al. (61). Free proline (fPro) concentrations were determined using the Ninhydrin chromogenic method (56). Soluble sugar content was determined via the anthrone colorimetric method (62).

#### Determination of plant Pb and Cd content

Oven-dried *B. juncea* root and leaf samples were ground into a fine powder. Then, 0.25 g of the powdered samples were mixed with 10 mL of a digestion solution composed of nitric acid (HNO₃) and perchloric acid (HClO₄) in a 3:1 (vol/vol) ratio. The mixture was left to digest overnight until the liquid volume reduced to approximately 1 mL. The digested samples were then diluted to a final volume of 25 mL with deionized water. After filtration, the concentrations of heavy metals were determined by atomic absorption spectrometry (PinAAcle 990T, PerkinElmer, USA).

#### Quantification of heavy metal transporter gene expression

Total RNA was extracted from fresh leaves using TRIzol reagent (New Cell and Molecular Biotech, Suzhou, China) according to the manufacturer’s instructions. RNA concentration and purity were assessed spectrophotometrically with a NanoDrop One/OneC (Thermo Fisher Scientific, China) (A260/A280 = 1.8–2.0, A260/A230 = 2.0–2.2) to ensure no protein or polysaccharide contamination. RNA isolation was performed for each replicate, with three replicates. Subsequently, 1 μg of total RNA was reverse-transcribed into cDNA using the All-in-One Script RT Premix kit (New Cell and Molecular Biotech, Suzhou, China) according to the manufacturer’s protocol, which includes a gDNA Eraser step to eliminate genomic DNA contamination. For quantitative real-time PCR (qRT-PCR) analysis, reactions were performed using 2× SYBR Green qPCR Premix (New Cell and Molecular Biotech, Suzhou, China) on an ABI QuantStudio 3 Real-Time PCR System (Applied Biosystems, Singapore). The reaction system (20 μL) included 10 μL of 2× SYBR Green Premix, 0.4 μL of each forward/reverse primer (10 μM), 1 μL of cDNA template (1:10 diluted), and 8.2 μL of nuclease-free water. The amplification program was 95°C for 30 s, followed by 40 cycles of 95°C for 10 s and 60°C for 30 s. The actin gene served as an internal control. Relative gene expression levels were calculated using the 2^(–ΔΔCT)^ method (63). The primer sequences used for RT-qPCR are listed in Table S1.

#### Data processing and statistical analysis

Means and standard deviations were calculated using Microsoft Excel (Microsoft Corp., WA, USA) and IBM SPSS Statistics version 26 (IBM Corp., NY, USA). Data normality and homogeneity of variance were assessed using the Shapiro-Wilk and Levene tests, respectively. Differences among treatment groups were analyzed by one-way analysis of variance (ANOVA), and when the ANOVA was significant, post hoc pairwise comparisons were conducted using Fisher’s least significant difference (LSD) test. The meanings of the annotations in the figures are explained as follows: “bars labeled with the same letter indicate no statistically significant difference between groups (*P* > 0.05, Fisher’s LSD test)” or “asterisks denote statistically significant differences from the control group: *P < 0.05, **P < 0.01, ***P < 0.001.” Correlation analysis and principal component analysis (PCA) were performed using OmicShare tools (https://www.omicstudio.cn/tool) with log-transformed data. To explore the relationships among soil bioavailable heavy metals, soil enzyme activities, soil nutrients, plant Pb/Cd concentrations, plant antioxidant systems, root traits, and photosynthetic parameters, partial least squares path modeling (PLS-PM) was applied (64), a method used to identify cause-and-effect relationships between observed and latent variables. Path coefficient estimates and coefficients of determination (*R²*) were estimated and their significance was assessed using the plspm package in R (v. 3.3.3) based on 1,000 bootstrap samples. Data visualization was carried out using Origin 2021, Adobe Illustrator 2019, and the online platform FigDraw (https://www.figdraw.com/#/).

## RESULTS

### Screening of Pb–Cd tolerant bacterial strains

**Fig 1:**
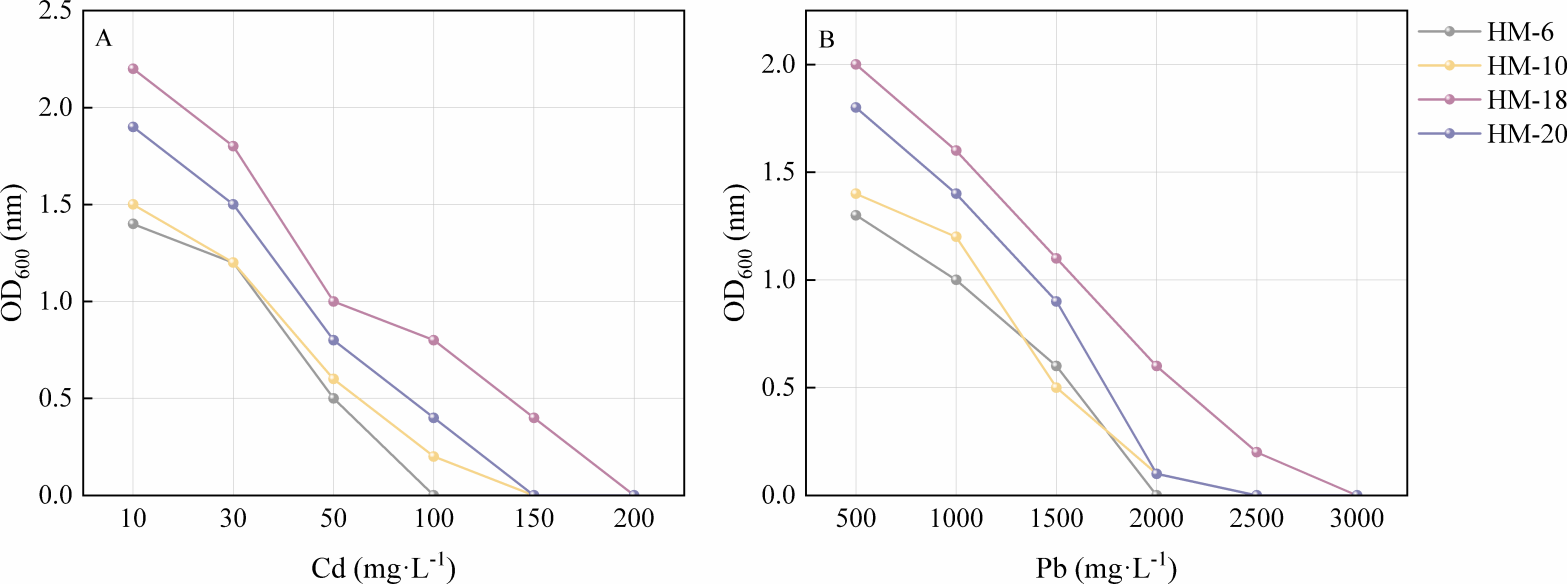
Minimum inhibitory concentrations (MICs) of CdCl_2_ (**A**) and PbCl_2_ (**B**) for four bacterial isolates. The MIC values for the four bacterial isolates designated HM-6, HM-10, HM-18, and HM-20 are shown in the figure. The MICs were determined by measuring the optical density (OD₆₀₀) at varying concentrations of CdCl₂ (10–200 mg·kg^−1^) and PbCl₂ (500–3,000 mg·kg^−1^).

A total of 40 bacterial strains were isolated and designated as HM-1 to HM-40. Heavy metal screening was conducted using concentrations of 500 mg·kg^−1^ Pb^2+^ and 10 mg·kg^−1^ Cd^2+^ as thresholds to assess microbial tolerance. We identified four bacterial strains (HM-6, HM-10, HM-18, and HM-20) showing strong tolerance to both Pb and Cd. They were further evaluated through tolerance growth experiments. The four strains showed different growth responses when cultured with various concentrations of Pb²^+^ and Cd²^+^ (Fig. 1). Each strain demonstrated differential sensitivity to the same heavy metal. Notably, strain HM-18 exhibited strong growth and high resistance under both Cd- and Pb-contaminated conditions. Its tolerance thresholds reached 150 mg·kg^−^¹ for Cd and 2,500 mg·kg^−^¹ for Pb.

### Pb and Cd mobilization capacity of Pb–Cd tolerant bacterial strains

**Fig 2:**
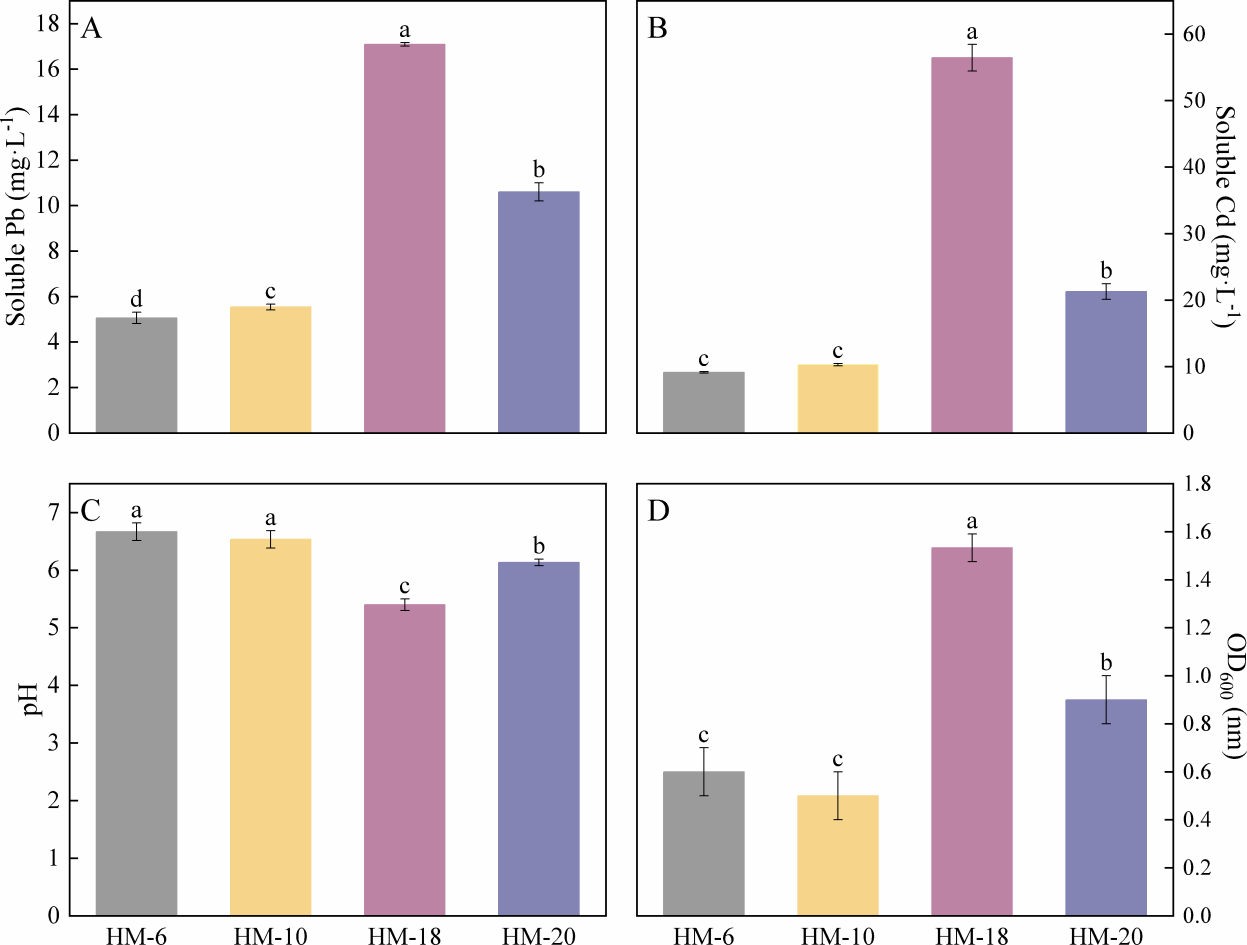
Soluble Pb (**A**), soluble Cd (**B**), pH (**C**), and OD_600_ (**D**) in cultures of different bacterial strains after 48-h incubation with 200 mg·L^−1^ PbCO₃ and 100 mg·L^−1^ CdCO₃. The results are presented as mean ± standard deviation (*n* = 3). Different lowercase letters indicate significant differences between treatments (*P* < 0.05).

Compared to the control group, the inoculation of HM-6, HM-10, HM-18, and HM-20 significantly increased the concentrations of water-soluble Pb and Cd in the culture solution after 48 h of incubation (Fig. 2). Among these, strain HM-18 demonstrated a significantly greater enhancement effect (*P* < 0.05) than the other three strains. After 48 h of incubation, the pH of the control medium was 6.90, whereas a decrease in pH was observed in all inoculated treatments. The HM-18 treatment caused a significantly greater pH reduction (*P* < 0.05) compared to the other three strains. Furthermore, after a 48-h incubation, the HM-18 treatment showed significantly higher soluble Pb and Cd levels in the culture medium compared with other treatments. These results indicate that the growth of strains HM-6, HM-10, HM-18, and HM-20 gradually solubilized Pb and Cd (from their insoluble forms), confirming their metal mobilization potential. HM-18 exhibited the highest mobilization capacity and was therefore selected for subsequent experiments. Strain HM-18 also demonstrated potassium-solubilizing capacity (10.47 mg·L^−1^) and siderophore production ability (43.69%).

### Identification of bacterial strain HM-18

**Fig 3:**
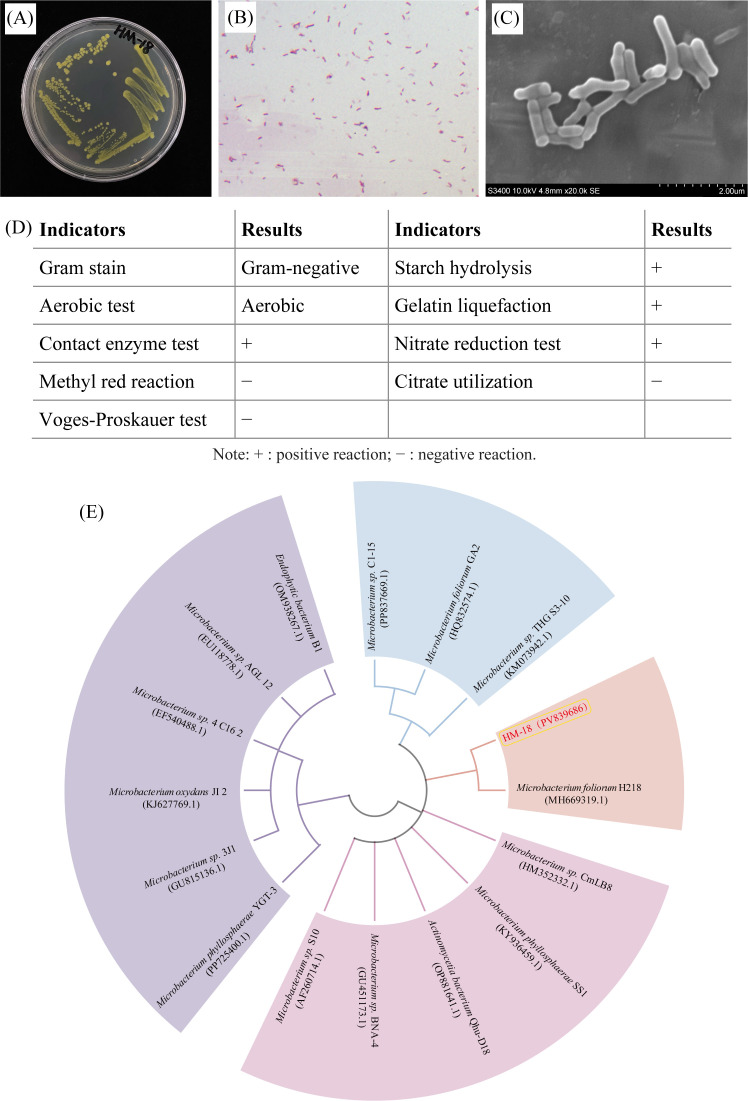
Identification of the strain HM-18. The colony morphology of strain HM-18 (**A**). Gram staining of strain HM-18 (**B**). Scanning electron micrograph of strain HM-18 (10,000× magnification) (**C**). Physiological and biochemical characteristics of strain HM-18 (**D**). Phylogenetic tree of strain HM-18 based on 16S rRNA gene sequences (**E**).

The surface of strain HM-18 was smooth, with a neat, opaque appearance and slightly yellowish edges (Fig. 3A). Gram staining revealed that HM-18 was a Gram-negative, irregular rod (Fig. 3B). Scanning electron microscopy analysis confirmed its rod-shaped morphology, with dimensions of approximately 2.28–3.00 µm in width and 6.12–16.20 µm in length (Fig. 3C). Biochemical tests indicated that HM-18 was negative for the Voges-Proskauer test, methyl red test, and citrate utilization. However, it tested positive for gelatin hydrolysis, contact enzyme activity, starch hydrolysis, and nitrate reduction (Fig. 3D). Phylogenetic analysis based on the neighbor-joining method revealed that the closest homologous strain to HM-18 was *Microbacterium foliorum* H218 (Fig. 3E). Based on a polyphasic analysis combining morphological, physiological, biochemical, and molecular data, strain HM-18 (accession number PV839686) was identified and classified as *Microbacterium foliorum*.

### Effect of HM-18 strain inoculation on Pb and Cd bioavailability in soil

**Fig 4:**
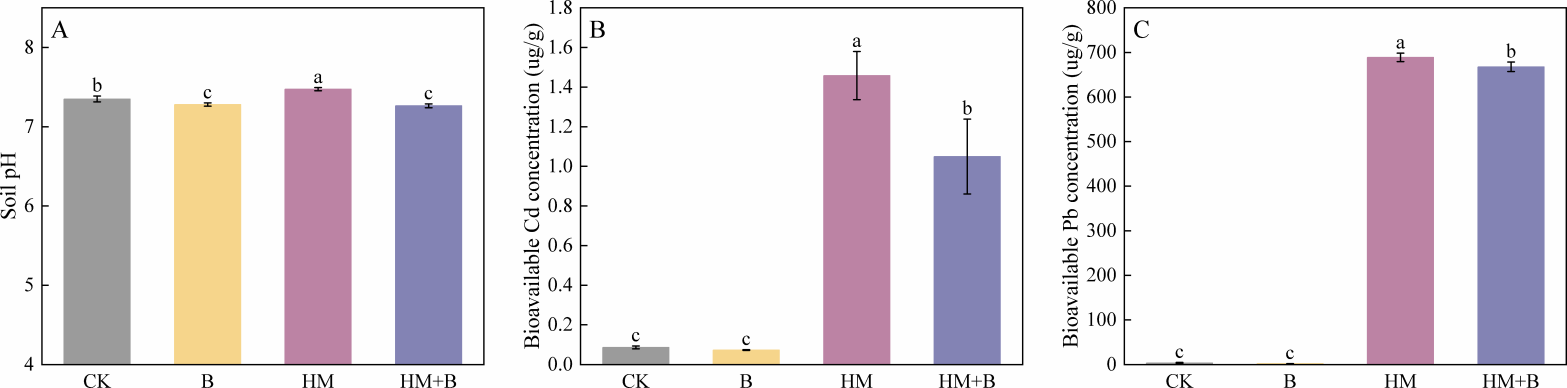
Effects of Pb–Cd mobilizing bacteria on soil pH and activation of insoluble Pb/Cd fractions. (**A**) Soil pH; (**B**) bioavailable Cd concentration; (**C**) bioavailable Pb concentration. The four treatments were CK (control, no heavy metals or bacteria), B (inoculated with *M. foliorum* only, no heavy metals), HM (heavy metals only, no bacteria), and HM+B (heavy metals with *M. foliorum* inoculation). The soils for the HM and HM+B treatments were spiked with 800 mg·kg^−1^ Pb and 80 mg·kg^−1^ Cd. The results are presented as mean ± standard deviation (*n* = 3), and different lowercase letters indicate significant differences between treatments (*P* < 0.05).

Inoculation with the Pb–Cd mobilizing bacteria significantly altered soil pH and promoted the transformation of insoluble Pb/Cd into more bioavailable forms (Fig. 4). Inoculation with HM-18 treatments consistently lowered soil pH, with significant reductions observed in both non-contaminated (B vs CK) and metal-contaminated conditions (HM+B vs HM) (Fig. 4A). Under heavy metal stress, the introduction of the mobilizing bacterium (HM+B treatment) significantly decreased the bioavailable Pb and Cd content in soil compared to the HM treatment alone (*P* < 0.05; Fig. 4B and C).

### Effects of HM-18 strain inoculation on soil nutrients and enzyme activities

**Fig 5:**
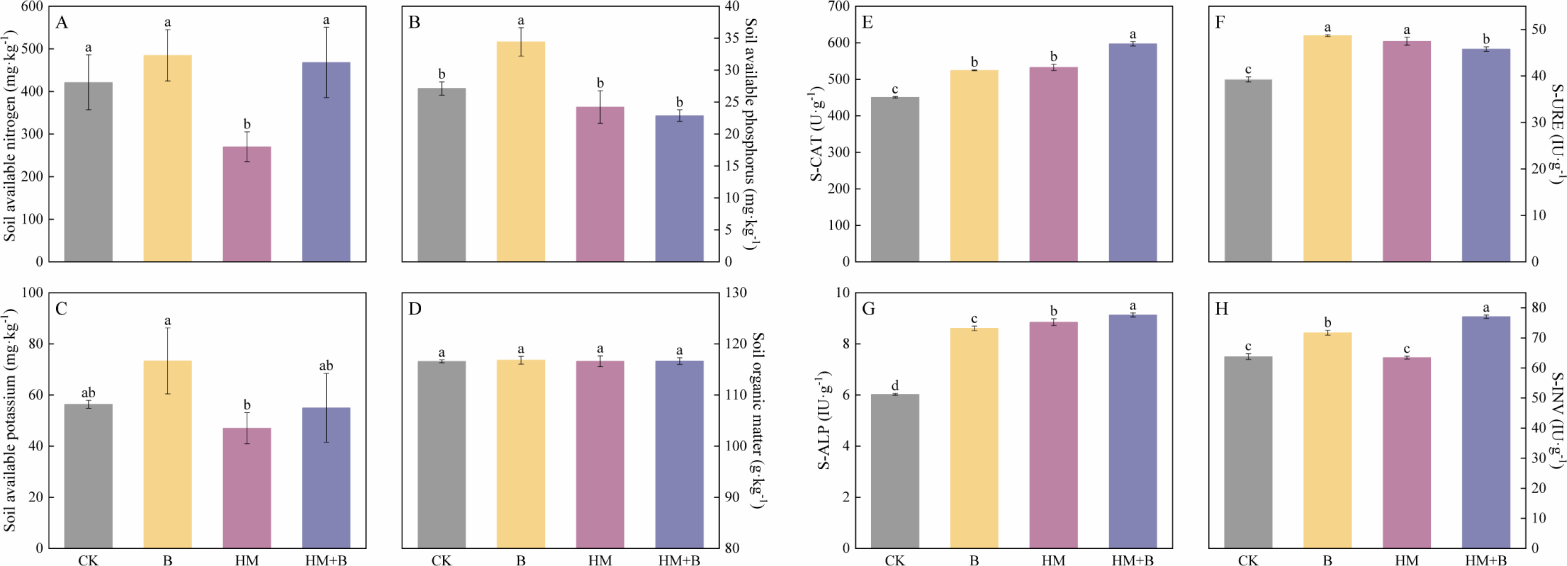
Effects of Pb–Cd co-contamination and inoculation with Pb–Cd mobilizing bacteria on soil available nitrogen content (**A**), soil available phosphorus content (**B**), soil available potassium content (**C**), soil organic matter content (**D**), S-CAT (**E**), S-URE (**F**), S-ALP (**G**), and S-INV (**H**) in soil cultivated with *B. juncea* (S-CAT, soil catalase; S-URE, soil urease; S-ALP, soil alkaline phosphatase; S-INV, soil invertase). The results are presented as mean ± standard deviation (*n* = 3), and different lowercase letters indicate significant differences between treatments (*P* < 0.05).

The combined treatment of Pb–Cd co-contamination and inoculation with Pb–Cd mobilizing bacteria altered soil nutrient availability. Compared to CK, treatment B significantly increased AP by 26.89% (Fig. 5B). In contrast, the HM treatment alone resulted in a 35.89% reduction in AN content (Fig. 5A). Notably, the combined HM+B treatment demonstrated a 73.30% increase in AN compared to HM alone (Fig. 5A). However, none of the treatments induced statistically significant changes in SOM content (*P* < 0.05).

Compared to CK, treatment B significantly increased all tested enzyme activities (S-CAT: +16.41%, S-URE: +23.96%, S-ALP: +42.87%, S-INV: +12.47%; *P* < 0.05). Similarly, HM treatment enhanced the activities of S-CAT (18.18%), S-URE (20.87%), and S-ALP (46.91%) (*P* < 0.05). In the combined HM+B treatment, enzyme activities responded differentially compared to HM alone: while the activities of S-CAT (12.16%), S-ALP (3.20%), and S-INV (21.46%) were significantly elevated (*P* < 0.05), that of S-URE activity was significantly reduced by 3.58% (*P* < 0.05).

### Effects of HM-18 strain inoculation on *B. juncea*

**Fig 6:**
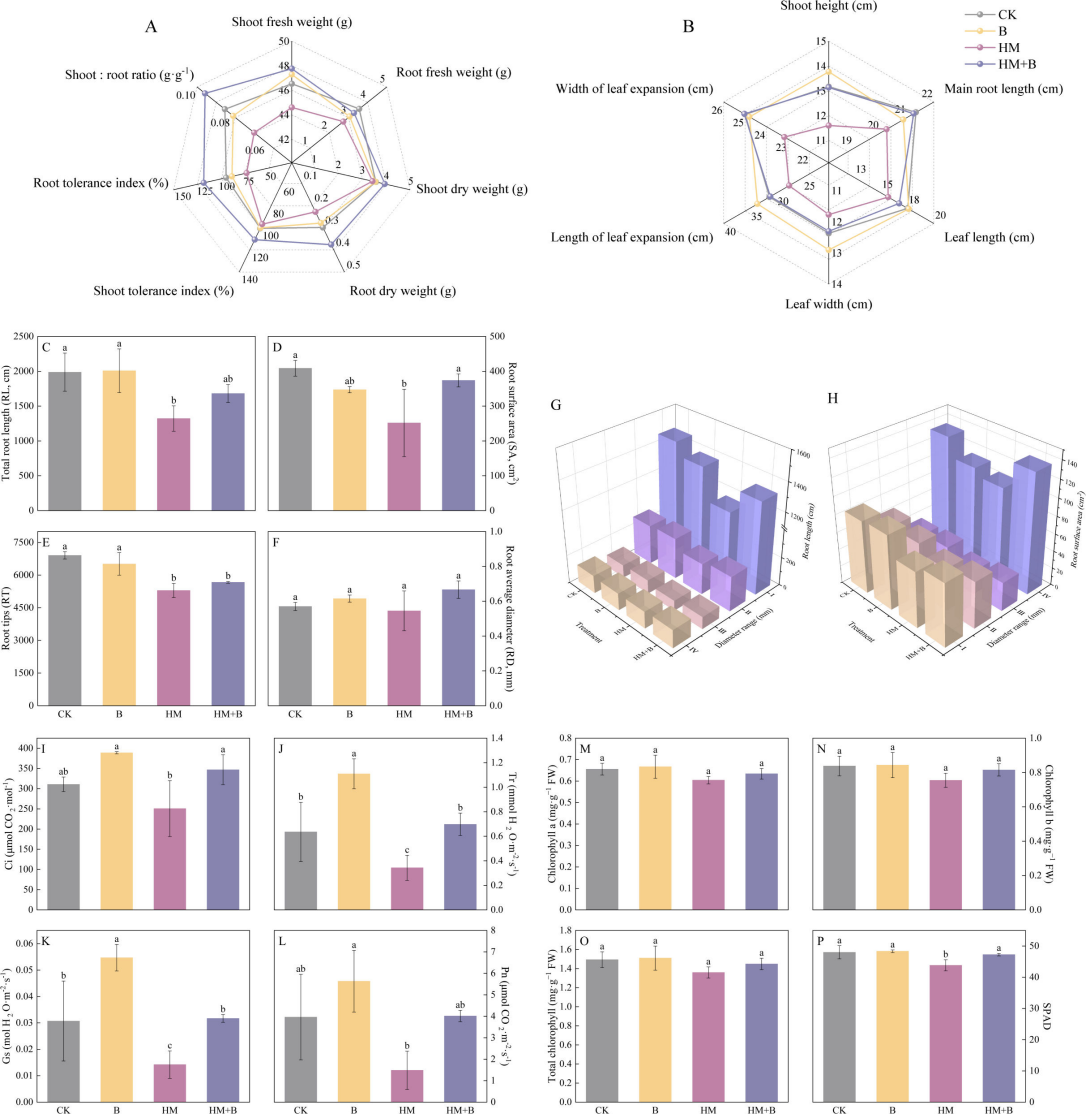
Effects of different treatments on the morphology, biomass, and photosynthesis of *B. juncea*. (**A**) Biomass and tolerance indices; (**B**) morphological parameters; (**C**) total root length; (**D**) total root surface area; (**E**) root tips; (**F**) root average diameter; (**G**) root length of different diameter classes; and (**H**) root surface area of different diameter classes. Roots were classified into four levels based on diameter (RD): class I (0 < RD ≤ 0.5 mm), class II (0.5 < RD ≤ 1.0 mm), class III (1.0 < RD ≤ 1.5 mm), and class IV (RD > 1.5 mm). (**I**) Ci; (**J**) Tr; (**K**) Gs; (**L**) Pn; (**M**) chlorophyll a; (**N**) chlorophyll b; (**O**) total chlorophyll; (**P**) SPAD (Ci, intercellular CO_2_ concentration; Tr, transpiration rate; Gs, stomatal conductance; Pn, photosynthetic rate). The results are presented as mean ± standard deviation (*n* = 3) and different lowercase letters indicate significant differences between treatments (*P* < 0.05).

We found that inoculation with the Pb–Cd mobilizing bacteria effectively enhanced both the biomass production and growth of *B. juncea* in Pb–Cd co-contaminated soil. Compared to CK, the HM treatment (Pb–Cd contamination alone) significantly decreased root dry weight (23.33%), fresh weight (22.91%), root tolerance index (23.33%), root-to-shoot ratio (20.35%), shoot height (11.90%), maximum leaf length (10.46%) and width (4.85%), as well as expansion length (11.91%) and width (7.33%) (*P* < 0.05) (Fig. 6A and B). In contrast, inoculation with Pb–Cd mobilizing bacteria in Pb–Cd co-contaminated soil (HM+B treatment) significantly increased shoot dry weight (14.66%), root dry weight (63.77%), shoot tolerance index (14.66%), root tolerance index (63.77%), root-to-shoot ratio (42.77%), plant height (13.22%), maximum leaf length (6.58%) and width (4.53%), and expansion length (12.92%) and width (8.06%) (*P* < 0.05) (Fig. 6A and B).

In addition, the HM treatment significantly inhibited root morphological development of *B. juncea* compared to CK, with reductions of 33.51% in total RL, 38.39% in root SA, and 23.36% in RT (Fig. 6C through E). Particularly, the HM treatment markedly suppressed fine root development, decreasing RL in diameter classes I–III by 20.78%, 21.22%, and 20.66%, respectively, with corresponding SA reductions of 23.35%, 22.60%, and 20.58%. However, the HM+B treatment effectively alleviated these inhibitory effects, resulting in a remarkable 48.47% increase in total SA. This improvement was primarily attributed to significant increases in RL for diameter classes I (14.56%) and III (19.47%), along with a 19.76% enhancement in SA for class III roots. Notably, RD remained stable across all treatments without significant differences (*P* < 0.05), suggesting that bacterial inoculation specifically promoted root elongation and surface expansion rather than altering root thickness under Pb–Cd co-contamination conditions (Fig. 6G and H).

Compared to CK, bacterial inoculation alone (B treatment) significantly enhanced photosynthetic performance, increasing Ci by 25.17% and Tr by 74.42% (*P* < 0.05). In contrast, the HM treatment markedly suppressed multiple photosynthetic parameters, reducing Tr by 46.04%, Gs by 53.80%, and SPAD values (an indicator of chlorophyll content) by 8.68% (*P* < 0.05). However, the HM+B treatment effectively mitigated these adverse effects, showing significant improvements in Ci (38.40%), Tr (103.25%), Gs (123.53%), and SPAD values (4.53%) relative to HM alone (*P* < 0.05). Although chlorophyll content followed a similar trend, the changes were not statistically significant (Fig. 6I through P).

### Effects of HM-18 strain inoculation on antioxidant defense system in *B. juncea* roots

**Fig 7:**
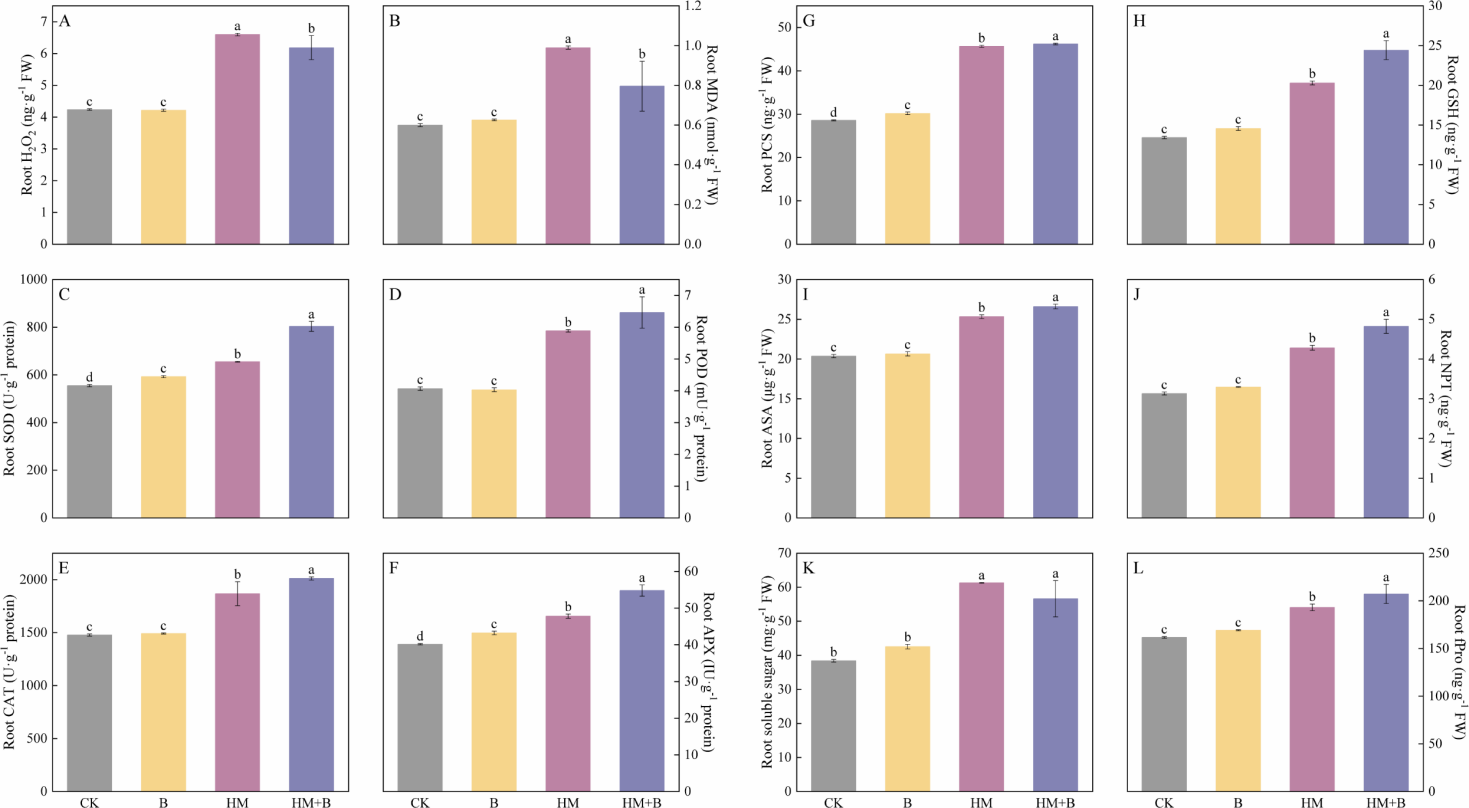
Effects of Pb–Cd co-contamination and inoculation with Pb–Cd mobilizing bacterial strains on oxidative stress markers, non-enzymatic antioxidants, and antioxidant enzyme activities in *B. juncea*. (**A**) H_2_O_2_; (**B**) MDA; (**C**) SOD; (**D**) POD; (**E**) CAT; (**F**) APX; (**G**) PCS; (**H**) GSH; (**I**) AsA; (**J**) NPT; (**K**) soluble sugars; and (**L**) fPro (MDA, malondialdehyde; SOD, superoxide dismutase; POD, peroxidase; CAT, catalase; APX, ascorbate peroxidase; PCS, phytochelatin synthase; GSH, reduced glutathione; AsA, ascorbate; NPT, non-protein thiols; fPro, free proline). The results are presented as mean ± standard deviation (*n* = 3). Different lowercase letters indicate significant differences between treatments (*P* < 0.05).

The levels of key oxidative stress indicators, MDA and hydrogen peroxide (H₂O₂), were significantly elevated in the roots of *B. juncea* under Pb–Cd co-contamination (HM treatment) compared to CK (*P* < 0.05; Fig. 7A and B). Inoculation with the Pb–Cd mobilizing bacteria (HM+B treatment) effectively mitigated this oxidative damage, leading to significant reductions in both H₂O₂ and MDA levels compared to the HM treatment alone (*P* < 0.05).

The activities of antioxidant enzymes in the roots were significantly altered by the treatments (Fig. 7C through F). Pb–Cd co-contamination (HM) significantly increased the activities of SOD, POD, CAT, and APX compared to CK. Notably, bacterial inoculation under metal stress (HM+B) significantly increased the activities of these enzymes compared with the HM group (*P* < 0.05).

Under the condition of co-contamination of Pb and Cd (HM), compared with CK, the contents of key antioxidants (PCS, GSH, ASA, NPT, soluble sugars, and fPro) all increased significantly (Fig. 7, *P* < 0.05). This up-regulation indicates a systemic antioxidant response to metal-induced oxidative stress. Bacterial inoculation under metal stress (HM+B) significantly increased the levels of all these antioxidants except for soluble sugars to a higher degree (*P* < 0.05).

### Effects of HM-18 strain inoculation on Pb and Cd accumulation in *B. juncea* plants and the expression of heavy metal transporter genes in roots

**Fig 8:**
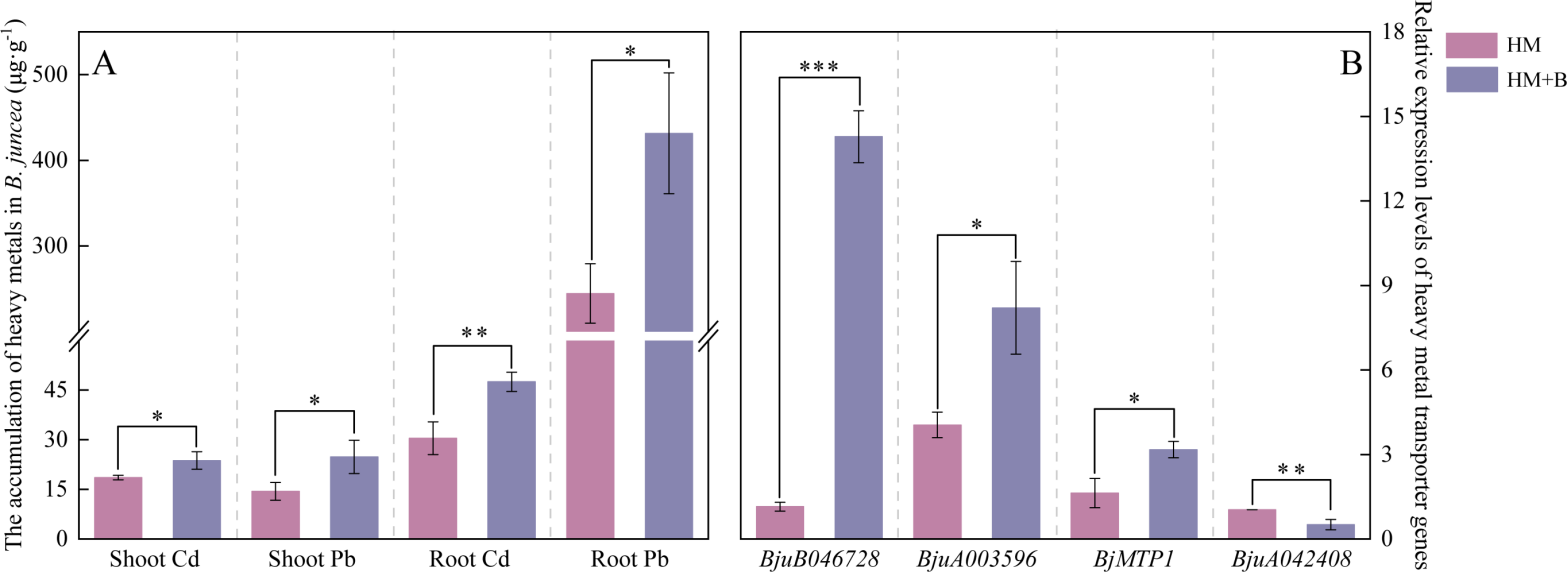
(**A**) Effects of Pb–Cd mobilizing bacteria on Pb and Cd accumulation in aboveground and belowground tissues of *B. juncea*. (**B**) Expression analysis of heavy metal transporter genes in *B. juncea* roots under Pb–Cd stress and inoculation with Pb–Cd mobilizing bacterial strains (qRT-PCR). Error bars represent standard deviation (*n* = 3). *P < 0.05, **P < 0.01, ***P < 0.001.

Inoculation with Pb–Cd mobilizing bacteria significantly enhanced metal accumulation in both aboveground (shoots) and belowground (roots) tissues of *B. juncea* under Pb–Cd co-contamination (Fig. 8A). Compared with the HM treatment, the HM+B treatment significantly increased Pb and Cd accumulation in *B. juncea* shoots by 72.38% and 27.61%, and in roots by 76.46% and 56.14%, respectively (*P* < 0.05). Roots accumulated higher concentrations of both metals than shoots.

To ensure the reliability of the qRT-PCR results for heavy metal transporter gene expression, the following key parameters were validated. (i) Specificity: melting curve analysis showed single, distinct peaks for all amplicons. (ii) Normalization: the actin reference gene exhibited stable expression across all treatments (CK, B, HM, and HM+B), with Ct values ranging from 22.1 ± 0.3 to 22.8 ± 0.4, ensuring valid normalization of target gene expression.

The expression level in CK was set to 1.0, and relative expression changes in other treatments were calculated accordingly. Gene expression analysis revealed significant differences in the expression of heavy metal transporter genes in the roots of *B. juncea* among treatments. Specifically, compared to the non-inoculated metal-stressed control (HM treatment), inoculation with the mobilizing bacterium HM-18 (HM+B treatment) significantly up-regulated the expression of *BjuB046728*, *BjuA003596,* and *BjMTP1* genes, while down-regulating the expression of *BjuA042408* gene (*P* < 0.05; Fig. 8B).

### Principal component analysis, Mantel test, Pearson correlation analysis, and PLS-PM analysis

**Fig 9:**
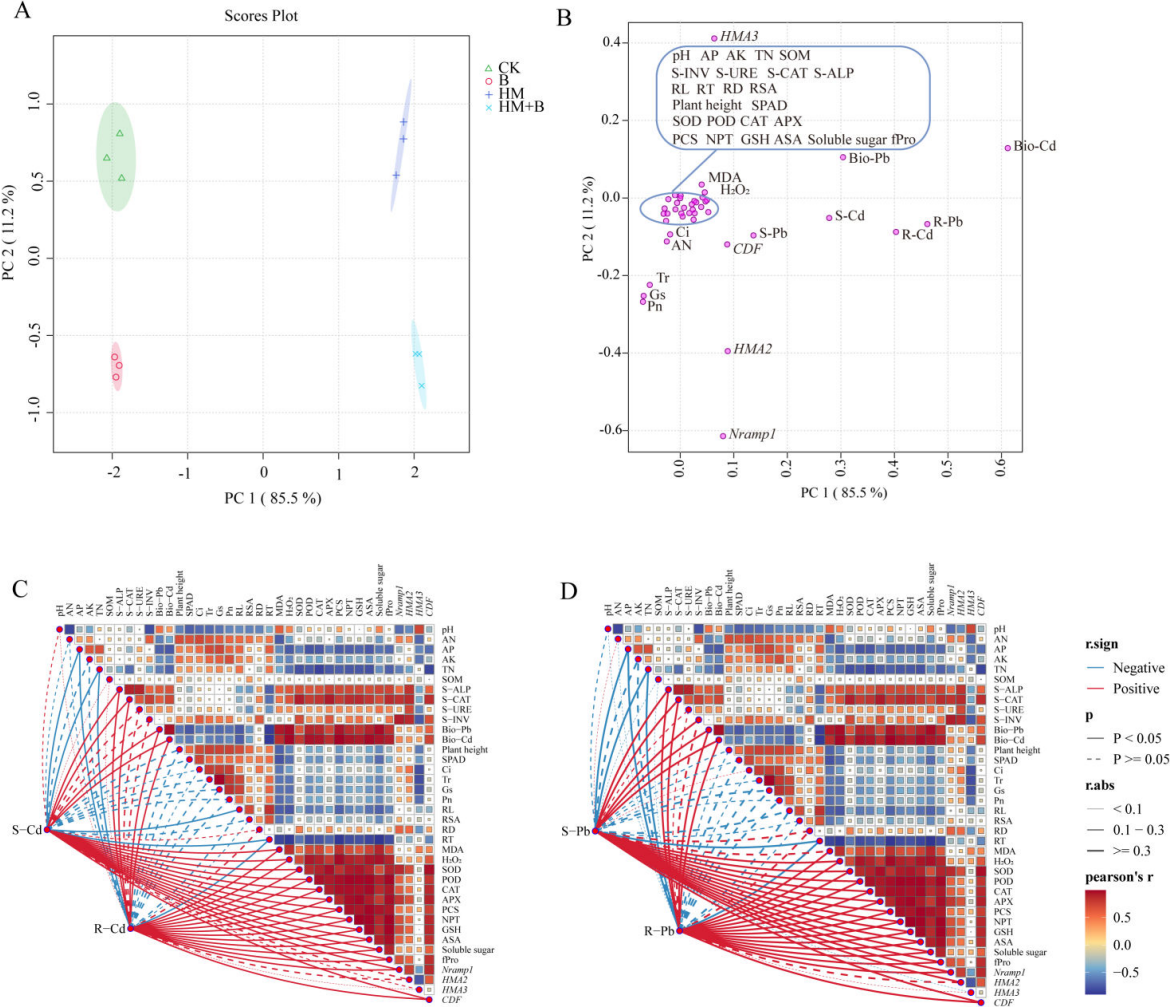
Principal component analysis and Cd/Pb accumulation patterns. Principal component analysis results are shown in (**A**) and (**B**). PC1 and PC2 denote the first and second principal components (variance explained in parentheses). Colored graphics represent treatment groups: CK (control, no heavy metals or bacteria), B (inoculated with *M. foliorum* only, no heavy metals), HM (heavy metals only, no bacteria), and HM+B (heavy metals with *M. foliorum* inoculation). Correlation heatmaps with network visualization for (**C**) Cd and (**D**) Pb accumulation, respectively. Color gradients reflect Pearson’s r (red/blue: positive/negative correlations). Solid and dashed lines indicate significance (*P* values), while line thickness reflects correlation strength (R).

PCA was performed to assess the integrated effects of Pb–Cd mobilizing bacterial inoculation and its relationship with Pb/Cd accumulation in *B. juncea* under metal stress (Fig. 9). The PCA revealed distinct clustering of the treatments, demonstrating significant treatment-dependent variations. Based on the PCA results, the indicators shown in Fig. 9 were selected for Mantel test and Pearson correlation analysis. Correlation analysis indicated that, across treatments, the following parameters were significantly positively correlated with Pb and Cd accumulation in plants (*P* < 0.05): root oxidative stress markers (MDA and H₂O₂); root antioxidant compounds (fPro, NPT, GSH, PCS, ASA, soluble sugar); root antioxidant enzyme activities (CAT, POD, SOD, APX); soil enzyme activities (S-ALP and S-CAT); soil bioavailable Pb and Cd concentrations; and the expression levels of root heavy metal transporter genes. Conversely, the following parameters showed significant negative correlations with plant Pb and Cd accumulation (*P* < 0.05): root morphological traits (RL and RT); soil nutrient indices (AP and TN); and photosynthetic parameters (SPAD value, Tr, and Gs) (Fig. 9).

**Fig 10:**
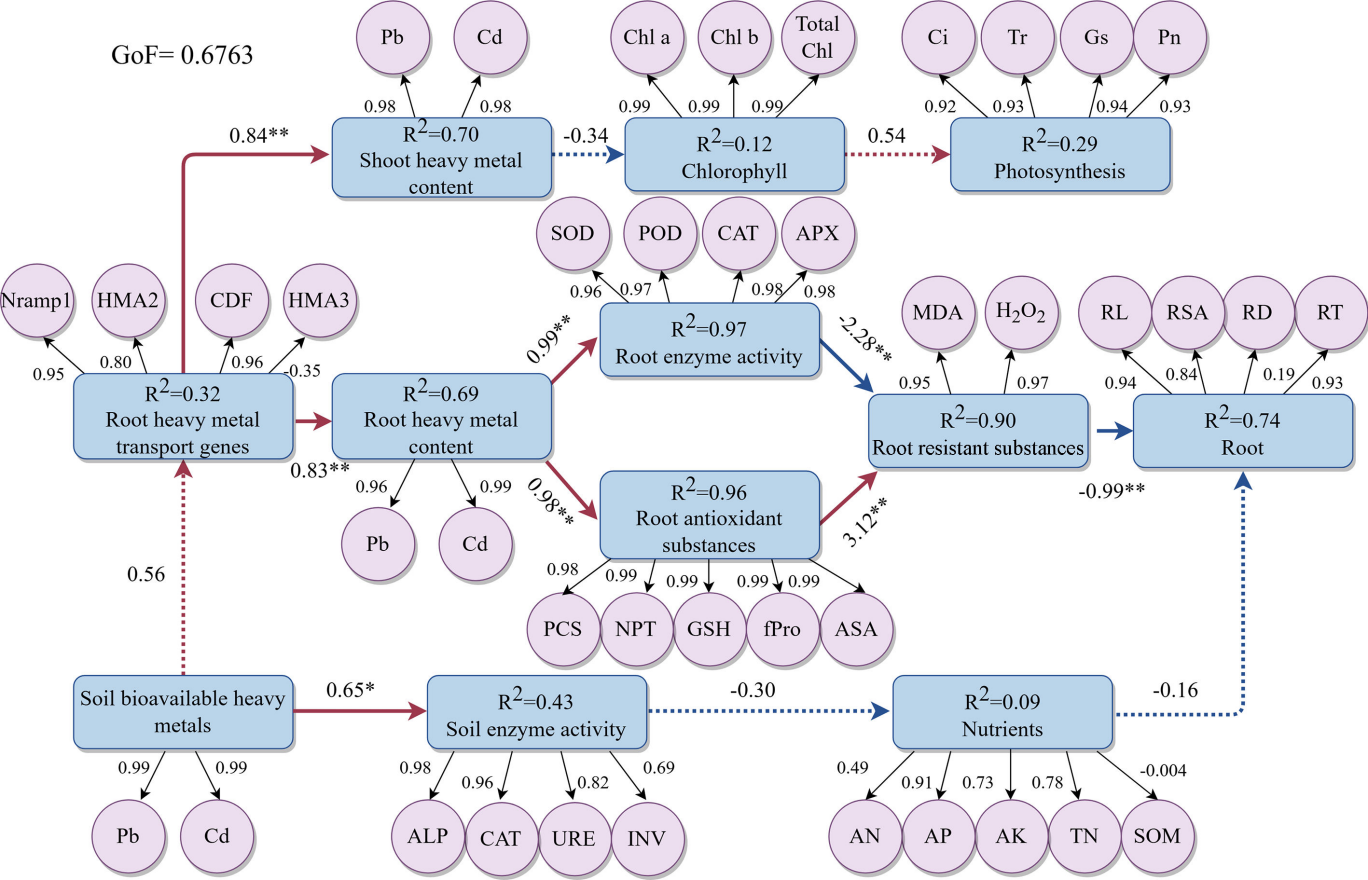
Relationships among bioavailable soil heavy metals, soil enzyme activities, soil nutrients, plant Pb/Cd contents, plant antioxidant systems, plant root traits, and plant photosynthetic parameters under inoculation with the Pb–Cd mobilizing bacterium HM-18, as analyzed by partial least squares path modeling (PLS-PM). Observed variables are represented by circles, and latent variables are represented by rounded rectangles. Path coefficients (between latent variables) and *R²* values (within ellipses) were calculated after 1,000 bootstrap iterations. Solid and dashed lines indicate significant (*P* < 0.05) and non-significant correlations, respectively, with red and blue colors representing positive and negative causal relationships. The goodness-of-fit (GoF) statistic was used to evaluate the overall model performance.

In this study, a conceptual model was constructed using PLS-PM to quantitatively evaluate the contributions of key factors and their interrelationships (Fig. 10). Within the PLS-PM framework, the value on an arrow leading from latent variables (depicted as rounded rectangles) to observed variables (represented as circles) corresponds to the loadings of the measured variables. The path coefficient (β) indicated on the arrow connecting the circles reflects the overall effect of the latent variables on the outcome. Positive β values denote a positive influence, while negative values signify an inhibitory effect. The magnitude of these numerical values indicates the strength of the respective effects. Notably, bioavailable Pb/Cd content in soil significantly influenced soil enzyme activity (β = 0.65, *P* < 0.05). The expression levels of heavy metal transporter genes in *B. juncea* roots exerted strong positive direct effects on both shoot Pb/Cd accumulation (β = 0.84) and root Pb/Cd content (β = 0.83). Furthermore, root Pb/Cd levels profoundly impacted both root antioxidant enzyme activities (β = 0.99) and non-enzymatic antioxidant content (β = 0.98). The non-enzymatic antioxidants demonstrated greater influence on anti-root substances (β = 3.12) compared to antioxidant enzymes (β = −2.28). Within this framework, the root system emerged as the primary regulatory node, with root resistant substances significantly affecting root physiology (β = −0.99).

## DISCUSSION

Four Pb–Cd tolerant bacterial strains were isolated from heavy metal-contaminated tailings. Among these, strain HM-18 was selected for further investigation due to its superior resistance profile. HM-18 showed the highest resistance to Pb and Cd, with MICs of 2,500 mg·L^−1^ for Pb and 150 mg·L^−1^ for Cd. In previous studies, *Bacillus cereus* and *Bacillus amyloliquefaciens* tolerated up to 2,000 mg·L^−1^ of Pb, while *Pseudomonas aeruginosa*, *Chryseobacterium* sp., and *Bacillus subtilis* tolerated 1,200 mg·L^−1^ each of Cd and nickel (Ni) (65). Jing et al. utilized Pb–Cd tolerant bacteria JYX10 and JYX7, isolated from the rhizosphere soil of bamboo in a mining area, to remediate Cd- and Pb-contaminated soil (66). They found that inoculation with these strains promoted the production of organic acids and siderophores around the roots of oilseed rape, which altered soil properties, including pH and other physicochemical characteristics, enhanced heavy metal bioavailability, and increased both biomass and heavy metal uptake in the plants. Extending this paradigm, our study not only confirms the role of siderophore production in metal mobilization by *M. foliorum* but also further elucidates its concomitant effects on soil enzyme activities and root architecture. In this study, strain HM-18 (*M. foliorum*) demonstrated both potassium-solubilizing and siderophore-producing capabilities. Previous studies have demonstrated that *M. foliorum* produces siderophores and synergistically interacts with plant root systems to effectively modulate arsenic (As) uptake and translocation (67).

We confirmed the capacity of *M. foliorum* in mobilizing Pb and Cd in contaminated soils by producing siderophores to enhance the bioavailability of Pb^2+^ and Cd^2+^ in soil (68, 69). Furthermore, inoculation with HM-18 also resulted in a significant decrease in soil pH, which is closely linked to heavy metal bioavailability. H^+^ ions and negatively charged ions form ion pairs that compete with heavy metal ions for adsorption sites (70), thereby increasing the mobility and bioavailability of heavy metals and facilitating subsequent phytoremediation efforts (71). These microbial metabolites also alter environmental acidity and form soluble chelates with Cd^2+^, preventing their precipitation and promoting the transfer of Cd from soil to plants (72, 73). In parallel, microorganisms herein act as essential “regulators” of geobiochemical processes, playing a vital role in maintaining soil vitality and ecological functions. The activities of URE, ALP, and CAT, which are sensitive indicators for assessing the extent of heavy metal pollution, can influence the cycling of C, N, and P in soil. However, these enzyme activities are also affected by various soil environmental conditions (74). HM-18 enhances soil acidification and K mobilization through mechanisms such as pH reduction, siderophore production, and the release of potassium-releasing metabolites. These processes decrease the binding of Cd^2+^ to microbial surface-active and sulfur groups, thereby increasing nutrient bioavailability and stimulating soil enzymatic activities (75, 76).

The morphological characteristics of the root system play a crucial role in a plant’s ability to explore soil resources and adapt to stressful environments (77). Following inoculation treatment, root system parameters showed an increase, which helped expand the roots' absorption area and enhance contact with the soil. Under nutrient-limited conditions, plants tend to increase root length and average diameter to improve their nutrient uptake capacity (78). Previous studies have demonstrated that arbuscular mycorrhizal fungi inoculation significantly enhances root parameters of *Ricinus communis* under Cr contamination, including root length, root surface area, and root tip number (79). This architectural expansion of the root system, induced by HM-18, thereby enhanced the plant’s capacity to access and absorb the mobilized heavy metals. Furthermore, heavy metal uptake serves as a vital parameter for predicting *B. juncea*’s capacity for Cd and Pb phytoextraction. Enhancing the bioavailability of heavy metals in soil primarily involves reducing the residual heavy metal content and converting metals into acid-soluble forms that are more readily absorbed by plants. In a study by Jeong et al., soil inoculation with phosphate-solubilizing bacteria, specifically *Bacillus megaterium*, led to the gradual dissolution of non-bioavailable and insoluble metal fractions, thereby increasing the mobility and bioavailability of Cd in the soil (80). In this study, HM-18 enhanced the bioavailability of Pb^2+^ and Cd^2+^ through mechanisms such as pH reduction and siderophore production. Additionally, the application of probiotics promoted plant growth, which further improved the overall efficiency of phytoremediation. It is noteworthy that the ratios of the contents of Pb and Cd in the root systems of the plants are higher than those in the aboveground tissues of the plants. The results obtained are consistent with the findings of Flores-Duarte, who demonstrated that alfalfa predominantly accumulated heavy metals in roots rather than in aerial parts (81). The enrichment level of heavy metals in plant roots is generally higher than in aboveground tissues, as metals are initially adsorbed by the roots before being translocated to the aboveground parts (82). The study demonstrated that, compared to HM treatments alone, inoculation with HM-18 significantly reduced the concentrations of plant-available Pb and Cd in the soil (Fig. 4), while markedly increasing metal accumulation in *B. juncea* (Fig. 8A). These findings indicate that plant uptake served as the primary sink for the mobilized metals.

Heavy metal toxicity has been shown to reduce transpiration and photosynthetic rates while increasing stomatal resistance (83). Several studies have demonstrated that plant-growth-promoting bacteria can enhance photosynthesis; for example, *Pseudomonas fluorescens* inoculation improved Pn, Ci, Tr, and Gs in *Sedum alfredii* (84), aligning with the findings of this study. Under heavy metal stress, total chlorophyll content, as well as chlorophyll a and chlorophyll b levels, typically decline (85). However, inoculation with HM-18 significantly increased these parameters, suggesting a protective effect. Previous research indicates that Cd stress suppresses pigment biosynthesis, disrupts carbon fixation, and ultimately reduces biomass yield (86, 87). Chlorophyll a is crucial for converting light energy into chemical energy, while chlorophyll b aids in light absorption and energy transfer (88). Elevated heavy metal levels can substitute magnesium in chlorophyll molecules, limiting chlorophyll synthesis, impairing electron transport, enzyme activity, and decreasing photosynthetic efficiency in the Calvin cycle (89). The presence of plant growth-promoting bacteria can mitigate these effects by increasing nutrient uptake through phosphate mobilization and secreting essential compounds necessary for chlorophyll synthesis, thereby enhancing light harvesting and photosynthesis (90). For instance, inoculation of *Klebsiella pneumoniae* in Vigna mungo increased chlorophyll levels under Cd stress (91). Similarly, Rizvi and Khan reported that supplementing Cu- and Pb-stressed Zea mays with *Azotobacter chroococcum* improved chlorophyll content, highlighting the beneficial role of PGPB in alleviating heavy metal-induced photosynthetic impairments (92).

ROS are essential components of plant responses to abiotic stresses. At appropriate levels, ROS participate in signal transduction pathways that regulate stress responses, but excessive ROS accumulation leads to oxidative stress, damaging cellular components (93). For plants, moderate amounts of H_2_O_2_ are beneficial for physiological processes; however, excessive H_2_O_2_, as a free radical, can harm cell membranes. MDA, a common product of membrane lipid peroxidation, reflects the extent of oxidative damage to cell membranes (94). Khan et al. found that high Ni treatment increased ROS levels in plants, but inoculation with *Bacillus megaterium* MCR-8 reduced the contents of MDA and H_2_O_2_ (95). Similar results were observed with HM-18 inoculation in the rhizosphere of *B. juncea*, where MDA and H_2_O_2_ levels decreased compared to non-inoculated plants, indicating HM-18’s role in alleviating oxidative damage. HM-18 enhances the plant’s antioxidant capacity mainly by regulating antioxidant enzymes and non-enzymatic components. Enzymes, such as CAT and APX, along with non-enzymatic antioxidants like AsA and GSH, are crucial for maintaining protein stability, structural integrity of cellular components, and reducing membrane lipid peroxidation (96, 97). This study demonstrates that HM-18 inoculation under heavy metal stress significantly boosts the activities of the antioxidant enzymes POD, SOD, CAT, and APX in *B. juncea*, thereby enhancing tolerance to Pb and Cd. These enzymes are vital for scavenging ROS and mitigating stress-induced toxicity. For instance, SOD converts superoxide radicals into H_2_O_2_, which is then detoxified by POD and CAT; POD alleviates H_2_O_2_ toxicity, while CAT primarily decomposes H_2_O_2_ into water (95). Zhang et al. reported that inoculation with PGP15 increased SOD and CAT activities, effectively reducing Cd stress (98). Similarly, Hou et al. observed that *Pogonatherum crinitum* enhances antioxidant enzyme activities, including POD and SOD, to scavenge ROS under increasing soil Pb levels (99). Likewise, Chandrasekhar and Ray highlighted the roles of SOD and CAT in *Eclipta prostrata*’s response to soil Pb stress, emphasizing the importance of antioxidant defense systems in plant heavy metal tolerance (100). Previous studies have demonstrated that under heavy metal stress, *alfalfa* employs APX to scavenge excess ROS, thereby mitigating oxidative stress-induced damage (101). This study found that the concentrations of AsA and GSH increased, thereby reducing the toxic effects of heavy metals on cells through the ASA-GSH cycle (102). In this study, proline content increased under Pb/Cd stress (HM and HM+B treatments) compared to the control, consistent with findings by John et al. on heavy metal (Cd and Pb) toxicity in *B. juncea* (103). Hayat et al. and Aslam et al. found that exogenously applied proline can enhance a plant’s ability to cope with heavy metal stress by boosting its endogenous proline levels (104, 105). This increase in proline plays a crucial role in managing oxidative stress by helping maintain intracellular redox homeostasis. Our results demonstrate that *M. foliorum* HM-18 plays a definitive role in orchestrating the antioxidant defense system of *B. juncea* under Pb–Cd co-contamination, a mechanism strongly supported by our PLS-PM analysis. The model revealed that root heavy metal content exerted a profound direct effect on both enzymatic (β = 0.99, *P* < 0.05) and non-enzymatic (β = 0.98, *P* < 0.05) antioxidant components, which aligns with the observed upregulation of these defenses upon HM-18 inoculation. Furthermore, the PLS-PM indicated a very strong determination (*R²* = 0.96) for the latent variable “root antioxidant substances,” which included AsA and GSH. This statistical robustness underpins our experimental finding that HM-18 markedly elevated the levels of these crucial non-enzymatic antioxidants. The concurrent increase in AsA and GSH is indicative of a reinforced ASA-GSH cycle, a central hub for H_2_O_2_ metabolism and redox homeostasis maintenance. Therefore, the strain HM-18 does not merely correlate with reduced oxidative stress; it actively primes a multi-faceted antioxidant system in the host plant, integrating both enzymatic scavenging and redox-buffering pathways to confer enhanced metal tolerance.

We found that inoculation with HM-18 under Pb–Cd stress can induce the upregulation of *Nramp1* (*BjuB046728*), *HMA2* (*BjuA003596*), and *CDF* (*BjMTP1*), and the downregulation of *HMA3* (*BjuA042408*). This may promote the transport of Pb and Cd from roots to leaves in *B. juncea* (Fig. 8B). Notably, the validity of this regulatory pattern is supported by rigorous qRT-PCR quality control, including specific amplification (single melting peaks and amplicons) and stable normalization with an internal control (actin gene), which eliminates potential confounding factors and ensures the observed gene expression changes reflect real biological responses rather than technical artifacts. Heavy metal ATPases (HMAs) are integral to metal transport in plants; they hydrolyze ATP to energize the transmembrane movement of metal ions (106, 107). Specifically, *HMA2* primarily mediates zinc (Zn) and Cd transport, playing a vital role in their translocation within the plant (108). *HMA3*, localized on the tonoplast membrane, predominantly facilitates the sequestration of metals, such as Cd, Zn, and Pb, into vacuoles, thus contributing to metal detoxification (109). The cation diffusion facilitator (*CDF*) family members are implicated in metal ion transport and resistance mechanisms in plants (110). NRAMP proteins are crucial during the uptake and internal movement of metal ions, with *Nramp1* notably involved in soil Cd uptake, as supported by multiple studies (111–113). Among these transporters, *HMA2* and *Nramp1*, both located on the plasma membrane, are key players in loading Cd into the xylem and facilitating root-to-shoot translocation (108, 112–114). The HM-18-induced up-regulation of *Nramp1*, *HMA2*, and *CDF*, validated by rigorous qRT-PCR, directly aligns with the enhanced Pb/Cd translocation from roots to shoots (72.38% Pb and 27.61% Cd increase in shoots) and total metal accumulation in *B. juncea* (Fig. 8A). This functional correlation further confirms that the gene expression changes are biologically meaningful. *Nramp1* promotes root uptake of Pb/Cd, *HMA2* facilitates xylem loading for root-to-shoot translocation, and *CDF* enhances metal efflux from cytoplasm (reducing cellular toxicity), while down-regulated *HMA3* reduces vacuolar sequestration (increasing metal availability for translocation). Consistent with this, a previous study has demonstrated that under heavy metal stress, *Ralstonia qingshengii* significantly upregulates *NRAMP1* and *HMA2* expression in *Sedum alfredii* (115).

Despite the promising results obtained in this controlled soil microcosm study, certain limitations and future research directions must be considered. The scalability and long-term stability of HM-18 inoculation under field conditions remain to be validated. Field environments introduce complex variables such as climate fluctuations, soil heterogeneity, and competition with indigenous microbial communities, which could affect the survival, activity, and efficacy of the inoculated strain. Moreover, the introduction of non-native microbes for bioremediation warrants a thorough assessment of ecological risks. Although HM-18 showed no pathogenic traits in our assays, its potential impact on the resident soil microbiome structure and function, such as unintended shifts in microbial diversity or disruption of ecological networks, was not investigated. Future research should include long-term field trials and employ metagenomic approaches to monitor the ecological impact of HM-18 inoculation, ensuring the development of safe and sustainable bioremediation strategies.

### Conclusion

In this study, we isolated *M. foliorum* HM-18, a strain exhibiting strong tolerance to Pb and Cd, as well as capabilities for Pb and Cd mobilization, potassium mobilization, and siderophore production. Soil microcosm experiments with *B. juncea* revealed that inoculation with HM-18 modulated the expression of critical heavy metal transport genes, promoted the translocation of Pb and Cd from roots to aerial parts, and thereby enhanced phytoremediation efficiency. Additionally, HM-18 inoculation improved soil enzyme activities, nutrient availability, and antioxidant defenses in plants, elevating antioxidant enzyme activities and substances, alleviating oxidative stress, and promoting healthier root development and photosynthetic capacity. These results underscore the potential of microbial agents like HM-18 in sustainable agriculture and environmental remediation. Our work contributes to a deeper understanding of microbe-plant interactions under heavy metal stress and highlights promising strategies for employing beneficial microbes in environmental management.

## Data Availability

The data that support the findings of this study are available from the corresponding author upon request. The raw reads in this work were deposited on NCBI (https://submit.ncbi.nlm.nih.gov) with the accession number PV839686.

## SUPPLEMENTAL MATERIAL

Graphical abstract spectrum.01964-25-s0001.tiff Visual depiction of the study.

Table S1 spectrum.01964-25-s0002.docx Primers for qRT-PCR analysis of heavy metal transporter genes.

ASM does not own the copyrights to Supplemental Material that may be linked to, or accessed through, an article. The authors have granted ASM a non-exclusive, world-wide license to publish the Supplemental Material files. Please contact the corresponding author directly for reuse.
