# Supplementary figures and images for: Identification of heavy metal-mobilizing bacteria and revealing of their mechanisms for bioremediation of Pb–Cd co-contaminated soils with Brassica juncea
Source: Microbiol Spectr. 2026 Mar 18;14(4):e01964-25. doi: 10.1128/spectrum.01964-25 (PMC13055314; doi:10.1128/spectrum.01964-25)

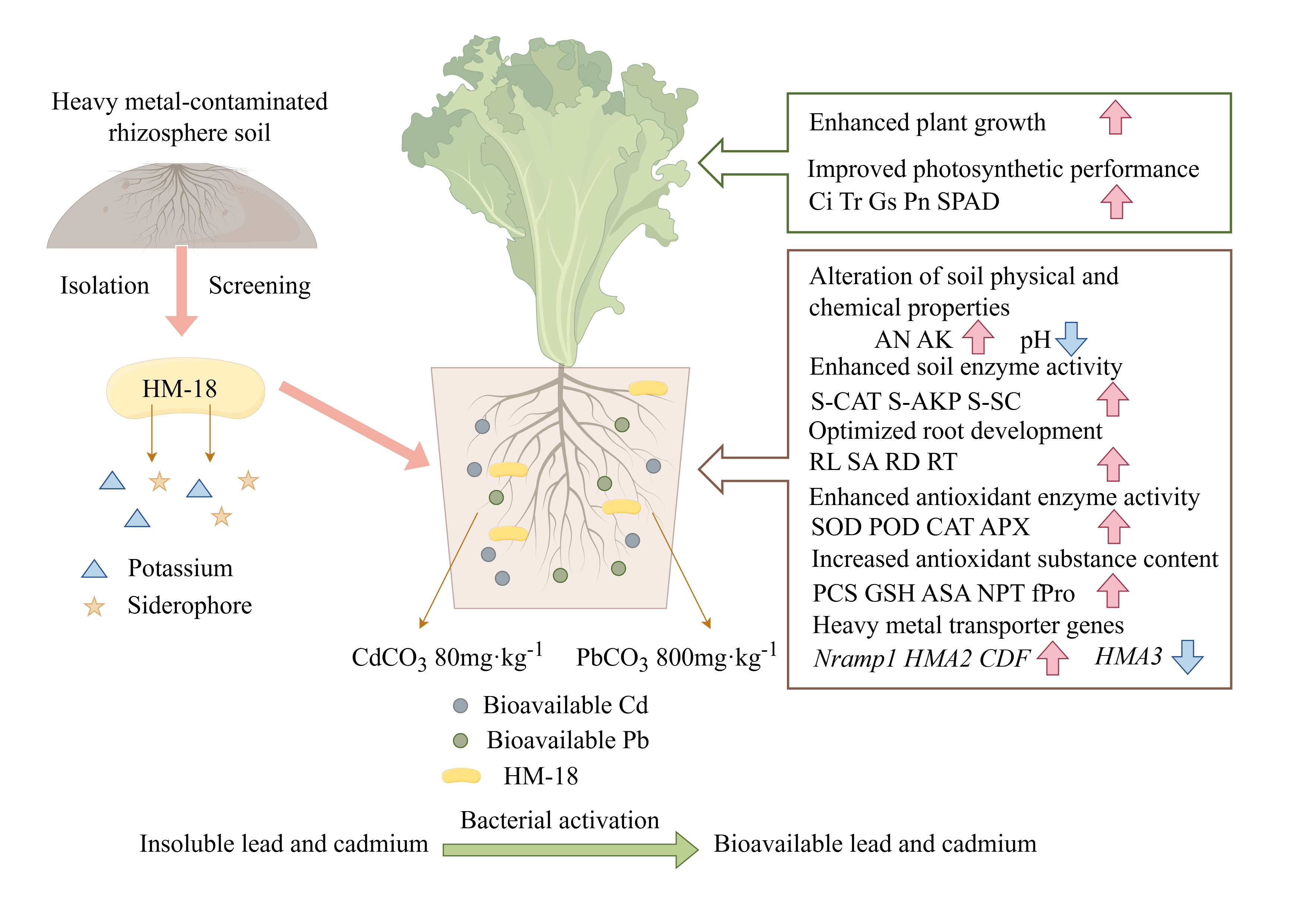

Supplement: Graphical abstract — Visual depiction of the study. [file spectrum.01964-25-s0001.tiff]
