# Supplementary material for: Identification of heavy metal-mobilizing bacteria and revealing of their mechanisms for bioremediation of Pb–Cd co-contaminated soils with Brassica juncea
Source: Microbiol Spectr. 2026 Mar 18;14(4):e01964-25. doi: 10.1128/spectrum.01964-25 (PMC13055314; doi:10.1128/spectrum.01964-25)
Supplement: Table S1 — Primers for qRT-PCR analysis of heavy metal transporter genes. [file spectrum.01964-25-s0002.docx]

Table S1. Primer for qRT-PCR analysis of heavy metal transporter genes

| Gene ID | Forward primer | Reverse primer |
| --- | --- | --- |
| *BjuB046728* | CTATGGCGGATGCTTCTGGA | ATCACCAAACCCGCACAAGA |
| *BjuA003596* | AAGAACCGTCATCGTCGTCCA | AGAAGTACGCCGGAAACCAC |
| *BjuA042408* | CAAGAACCGTCATCGTTGTCC | GCAACAAGAGCGAACCACT |
| *BjMTP1* | TGCGGCTTCTCAGATCTCAA | TGCGCATGGAGGCATTG |
| *Actin* | TGAAACCTTCGAATGCCCAG | GATTGGAACCGTGTGGCTCA |
